# Comparing the Effectiveness of Open and Minimally Invasive Approaches in Coronary Artery Bypass Grafting: A Systematic Review

**DOI:** 10.3390/clinpract14050147

**Published:** 2024-09-10

**Authors:** Arwa Alsharif, Abdulaziz Alsharif, Ghadah Alshamrani, Abdulhameed Abu Alsoud, Rowaida Abdullah, Sarah Aljohani, Hawazen Alahmadi, Samratul Fuadah, Atheer Mohammed, Fatma E. Hassan

**Affiliations:** 1 Department of Medicine and Surgery, Batterjee Medical College, Jeddah 21442, Saudi Arabia; 140065.ghadah@bmc.edu.sa (G.A.); 140011.abdulhameed@bmc.edu.sa (A.A.A.); 140238.rowaida@bmc.edu.sa (R.A.); 140321.sarah@bmc.edu.sa (S.A.); 140034.samratul@bmc.edu.sa (S.F.); 140278.atheer@bmc.edu.sa (A.M.); 2 Department of Medicine and Surgery, Vision College, Jeddah 23643, Saudi Arabia; 202313034@vision.edu.sa; 3 Faculty of Medicine, Taibah University, Al-Madinah Almunawwarah 41477, Saudi Arabia; tu4354677@taibahu.edu.sa; 4 Medical Physiology Department, Kasr Alainy, Faculty of Medicine, Cairo University, Giza 11562, Egypt; fatma.hassan@bmc.edu.sa; 5 General Medicine Practice Program, Department of Physiology, Batterjee Medical College, Jeddah 21442, Saudi Arabia

**Keywords:** minimally invasive CABG, open CABG, hospital stay, wound infection, mortality, angina recurrence

## Abstract

Coronary artery bypass grafting (CABG) is an essential operation for patients who have severe coronary artery disease (CAD). Both open and minimally invasive CABG methods are used to treat CAD. This in-depth review looks at the latest research on the effectiveness of open versus minimally invasive CABG. The goal is to develop evidence-based guidelines that will improve surgical outcomes. This systematic review used databases such as PubMed, MEDLINE, and Web of Science for a full electronic search. We adhered to the PRISMA guidelines and registered the results in the PROSPERO. The search method used MeSH phrases and many different study types to find papers. After removing duplicate publications and conducting a screening process, we collaboratively evaluated the full texts to determine their inclusion. We then extracted data, including diagnosis, the total number of patients in the study, clinical recommendations from the studies, surgical complications, angina recurrence, hospital stay duration, and mortality rates. Many studies that investigate open and minimally invasive CABG methods have shown that the type of surgery can have a large effect on how well the patient recovers and how well the surgery works overall. While there are limited data on the possible advantages of minimally invasive CABG, a conclusive comparison with open CABG is still dubious. Additional clinical trials are required to examine a wider spectrum of patient results.

## 1. Introduction

Coronary artery bypass grafting (CABG) is a frequently conducted surgical intervention aiming at restoring blood flow to the heart in individuals with coronary artery disease (CAD) [1]. CAD is a global health threat associated with substantial illness and death [2]. CAD, a prevalent cardiovascular disorder, frequently presents with distinctive indicators that warrant careful consideration. These indicators include symptoms such as angina, chest pain, or pressure, which may occasionally extend to the arms, neck, or jaw. In addition, dyspnea experienced during physical activity or even during periods of rest indicates insufficient blood flow to the heart. It is important to note that symptoms might vary among individuals; therefore, it is important to pay attention to both common indicators and unusual presentations [3].

There are many different types of CABG, each made to meet the specific needs of a patient and help them deal with the challenges of CAD. Historically, CABG has been conducted via an open technique called a sternotomy, a vertical incision in the chest that provides extensive access to the heart and its blood vessels [4]. The procedure has a proven history of success, demonstrating the ability to produce durable grafts and provide long-lasting relief from angina, reducing the likelihood of recurrent cardiac episodes [5]. This method revolutionized the treatment of advanced CAD [6]. However, ongoing progress has led to minimally invasive techniques in CABG, which offer less invasive options with potentially improved recovery times. Endovascular therapies, such as percutaneous coronary procedures, have also come up as other ways to control CAD. This has increased therapy options and made patient care better [7].

The development of minimally invasive techniques such as Minimally Invasive Direct Coronary Artery Bypass (MIDCAB) and Off-Pump Coronary Artery Bypass (OPCAB) has significantly altered the surgical paradigm. MIDCAB is the utilization of smaller incisions, also known as “keyhole” incisions, with a specific focus on addressing single-vessel disease [8]. Nevertheless, in recent times, minimally invasive methods have become increasingly popular because of their potential advantages, including decreased surgical trauma, shorter hospital stays, and quicker recovery. These benefits make them attractive to patients needing less invasive CAD management alternatives [9]. When dealing with CAD, which is very delicate, both traditional open-heart surgeries and the new minimally invasive treatments have their pros and cons [10].

This systematic review critically evaluates how well open and minimally invasive methods work for CABG. Although much research has examined the results of various processes, there is still a continuing dispute about their superiority. Proponents of the open technique contend that it offers enhanced visualization and accessibility to the heart, leading to enhanced rates of graft patency and long-term results. On the other hand, proponents of the minimally invasive method highlight the benefits of smaller cuts, such as decreased blood loss and postoperative pain, resulting in faster healing and higher patient contentment. This review aims to compare the results and complications of minimally invasive CABG to traditional CABG achieved through a sternotomy. Because CAD is a major global health issue that causes a lot of illness and death, the study aims to determine whether minimally invasive cardiac surgery (MICS) CABG can be better than traditional open CABG while also having lower hospitalization duration and mortality rates.

## 2. Materials and Methods

### 2.1. Search Strategy

The systematic review was registered in PROSPERO (CRD42024506685) and was conducted following the Preferred Reporting Items for Systematic Reviews and Meta-Analyses (PRISMA) guidelines [11] (Appendix D and Appendix E). A comprehensive electronic search was conducted using the following databases: PubMed, MEDLINE, and Web of Science, with no specific time frame. A search strategy has been developed by the authors F.E, A. A and approved by the rest of the research team. Studies related to the comparative effectiveness of open and minimally invasive approaches in CABG were identified inclusively using a combination of Medical Subject Headings (MeSH) such as “Coronary Artery Bypass Grafting” OR “Coronary Artery Bypass Surgery” OR “Coronary Artery Bypass” OR “Aortocoronary Bypass” OR “Bypass Surgery” OR “CABG” AND “Coronary Artery Disease” OR “Aortocoronary” OR “Open heart surgery” OR “Minimally invasive surgery” OR “Endoscopic Surgery” AND “Mortality” OR “Wound Infection” OR “Bleeding Rates” OR “Stroke rates” OR “Length of hospital stay”. To identify any missing articles, a further review of the references to the studies was conducted.

The search technique included searching several databases: PubMed (*n* = 3234), MEDLINE (*n* = 1202), and Web of Science (*n* = 2581). At first, the records were checked for duplicates, leaving 7017 distinct records. During the eligibility phase, 1053 records were reviewed, and 5964 records were eliminated based on established criteria. Out of the records reviewed, 174 full-text articles were evaluated for eligibility, excluding 879 articles with indicated reasons. Seventy-one papers met the criteria for inclusion in the qualitative synthesis.

### 2.2. Study Selection

#### 2.2.1. Inclusion Criteria

This review included studies that compare the effectiveness of open and minimally invasive approaches in coronary artery bypass grafting. This review considered various research designs, including randomized controlled trials (RCTs), quasi-experimental studies, cohort studies, case-control studies, and observational studies published in English. Furthermore, this review only included studies that were published in peer-reviewed journals or other credible sources.

#### 2.2.2. Exclusion Criteria

This systematic review excluded studies that do not investigate the effectiveness of open or minimally invasive approaches in CABG. We also excluded studies that focused on surgical procedures or interventions unrelated to CABG, animal studies, in vitro studies, and review articles. Studies published in languages other than English or with insufficient data, such as those that lack detailed outcome measures, specific numerical results, or relevant statistical analyses necessary to evaluate the efficacy of the surgical techniques, were not considered for inclusion in this review.

#### 2.2.3. Screening and Data Extraction

After conducting the primary search, the records were imported to Google Drive (Mountain View, CA, USA: Google) and Mendeley Desktop (Mendeley Ltd., London, UK), where duplicate articles were removed. The remaining results were then imported into Rayyan [(https://www.rayyan.ai/) accessed on 3 October 2023] for screening by three authors (R.A., S.A., H.A.) based on relevance determined by titles and abstracts. Next, the full texts of the studies that passed the initial screening were reviewed by two authors (S.F., A.M.) for the final inclusion or exclusion decision [12]. Any disagreements during the screening process were resolved through discussion with (A.A.) and the other researchers. Data were extracted from the selected studies through an Excel sheet, including the title, author’s name, country, year of publication, name of the journal, study design, level of evidence, sample size, surgical complication (wound infection rates, bleeding rates, and stroke rate), angina recurrence, length of hospital stay, and mortality rates.

#### 2.2.4. Quality Assessment and Bias Evaluation

We evaluated the included studies for their quality and potential bias using the Grading of Recommendations Assessment, Development, and Evaluation (GRADE) system. This comprehensive assessment revealed varying levels of evidence within the included studies, thereby offering valuable insights into their overall quality and potential sources of bias. Retrospective and prospective cohort studies used the Newcastle–Ottawa Scale for bias assessment (Appendix A). Additionally, we used the revised Cochrane risk-of-bias tool for randomized trials (RoB 2) to assess the risk bias of RCTs (Appendix B). We also used the MINORS tool to evaluate the quality of the non-randomized studies included in this review (Appendix C). These evaluations provide insights into the overall quality and potential sources of bias in the included studies, enhancing the robustness and reliability of the reported results.

### 2.3. Data Synthesis

Despite conducting a basic descriptive statistical analysis using Review Manager version 5.4.1 (Cochrane, London, UK), we could not conduct a meta-analysis because of the high heterogeneity and lack of consistent data formats in the included studies. The following aspects of the heterogeneity were visible as shown in Table 1 and Table 2: first, the variability in research methodologies encompassing randomized controlled trials (RCTs), cohort studies, and observational studies. Second, there are distinctions between traditional and minimally invasive procedures for CABG. Lastly, there is inconsistent reporting of outcomes such as wound infection rates, bleeding rates, stroke rates, death rates, and length of hospital stay.

## 3. Results

**Figure 1:**
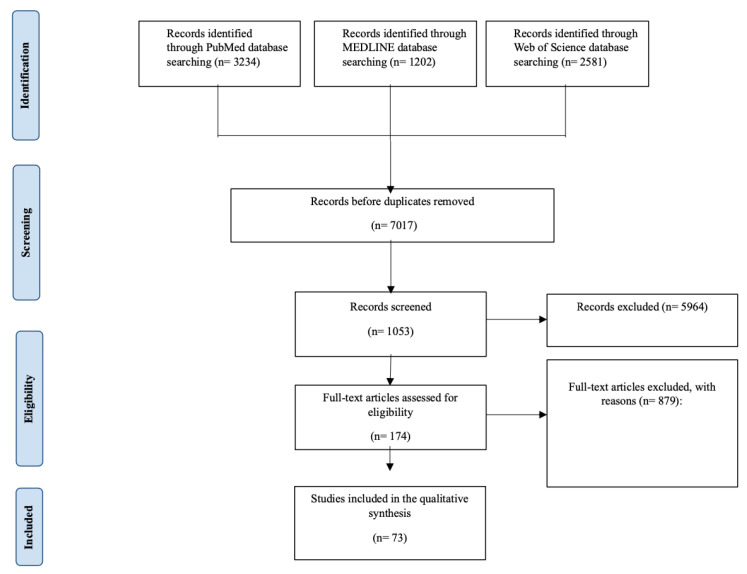
Detailed PRISMA chart used for this systematic review, outlining the many stages of this study’s selection process.

In our search, a total of only 73 articles fulfilled the full scrutiny required to be included in this systematic review [13,14,15,16,17,18,19,20,21,22,23,24,25,26,27,28,29,30,31,32,33,34,35,36,37,38,39,40,41,42,43,44,45,46,47,48,49,50,51,52,53,54,55,56,57,58,59,60,61,62,63,64,65]. A total of 879 articles were excluded from full-text review for many reasons. These exclusions were due to reasons such as not having the complete text available, duplicates, methodological shortcomings, or flawed results attributed mainly to different outcomes sought among studies included (diverse treatment strategies) and for research articles that contained diabetic patients but had no specific measurements of periodontal therapy within it. Other excluded papers were in a language other than English. A mix of research designs was observed across the included studies in this systematic review. The range of designs allowed for a more complete evaluation of the efficacy and approaches to CABG in open form or minimally invasive. The PRISMA flowchart of the systematic review is illustrated in Figure 1 and details all components. The 73 studies included in the systematic review were from several locations worldwide. The included studies were published from 1997 to 2023.

The articles that reference the comparative effectiveness of open and minimally invasive approaches in CABG, together with a comprehensive overview of the demographics of their participants, are detailed in Table 2.

### 3.1. Patients’ Profiles and Characteristics

A total of 60,954 patients were included in this systematic review. A total of 46,379 patients underwent open CABG and 14,575 minimally invasive CABGs. The age of participants differed significantly across studies, with mean ages 54 years (range for young group = 34–46 to old group = 75–97).

The analysis of the gender distribution across studies showed predominant involvement among males, with 40,187 participants compared with a total of 20,767 females. Our original research included a cohort of 102 patients with one-vessel disease of the LAD coronary artery, studied from December 1996 to December 1998 [16]. This study’s findings revealed significant benefits associated with using OPCAB. The OPCAB group had a 0% operative death rate, but the surgical time was significantly shorter than that of the MIDCAB group (4% mortality). These results suggest that OPCAB has a better technical result (*p* = 0.004). Recurrent angina occurred in 40% of MIDCAB and 27% of OPCAB patients during a mean follow-up period of 5.2 years [13]. Another study illustrated the rationale for the use of MICS as opposed to a traditional CABG trial. The trial aimed to include eighty-eight patients per group, using the SF-36 questionnaire as the primary tool for assessing quality of life (QoL) after a month. On the other hand, previous studies demonstrated that an average hospital stay following MICS is 5 days, while it takes at least nine more days in sternotomy CABG [18]. The Society of Thoracic Surgeons maintained a regional database for clinical quality improvement, from which a second study extracted patients with CABG. There was a total of 278 open CABG and 139 MICS CABG patients. In addition, the rates of serious morbidity were similar between matched groups, with an open CABG rate of 7.9% and a MICS CABG rate of 7.2%, respectively (*p* = 0.795). On a notable note, MICS CABG was associated with the advantages of less use of blood product transfusions (12.2% in the MICS CABG vs. 22.3% in open CABG; *p* = 0.013) and a shorter duration hospitalization period (6 days for MIS-CABG vs. 7 days for open CABG, *p* = 0.005). Furthermore, the findings demonstrated that patients who had MICS CABG had lower hospital charges, with a median of $27,906 vs. $35,011 for open CABG (*p* = 0.001) [35].

### 3.2. Patient-Reported Outcomes and Complications

This section provides an overview of the results of patient-reported outcomes and the incidence of complications related to open and minimally invasive techniques in CABG. From April 2008 to July 2011, a study was conducted that included a total of 74 patients in the MIDCAB group and 78 patients in the OPCAB group. The comparison between the two groups was deemed adequate based on the patient demographics and EuroSCORE values. The OPCAB had a lower rate of cerebrovascular accidents (1.3% for OPCAB vs. 1.4% for MIDCAB, *p* = 1.0), recurrent myocardial infarctions (0% for OPCAB vs. 1.4% for MIDCAB, *p* = 0.3), and wound infections (2.7% for OPCAB vs. 5.4% for MIDCAB, *p* = 0.4) [55].

## 4. Discussion

We conducted a systematic review to determine the differences in outcomes between minimally invasive and traditional open CABG procedures. We found a strong association between the type of surgery performed and surgical trauma, duration till recovery, and postoperative complications. Historically, they have performed MIDCAB and OPCAB earlier than Lima LAD. These provide clinical relevance to minimally invasive approaches, especially for patients with specific comorbidities who might not be able to recover from this extensive surgery if performed openly. This further supports the increased use of less-invasive techniques that have minimal physiological consequences while still providing good anterograde coronary perfusion.

Our review underscores that the decision to recommend open or minimally invasive CABG should be patient-specific [13,24]. Patient age, left ventricular ejection fraction (LVEF), and concomitant comorbidities such as chronic kidney disease (CKD) are all associated with outcomes. For instance, younger patients with fewer comorbidities are likely to benefit more from the use of minimally invasive techniques, as these approaches have demonstrated lower hospital and long-term mortality rates. Conversely, in patients with more complicated pathology, the traditional ‘open’ approach may still be necessary to achieve a good long-term outcome [66,67,68]. 

Data presented in our systematic review suggest that the immediate decisions for surgical intervention in CABG should be patient-centered and driven more by clinical indications to achieve optimal outcomes [26]. While the MICABG has demonstrated potential in this field, particularly for simpler cases and associated evaluations, traditional open CABG remains the gold standard for providing durable grafts that realistically promise excellent long-term symptom relief despite its more invasive nature. In turn, a minimally invasive approach should be considered for patients who are hemodynamically stable with multivessel coronary artery disease, and this group may offer a better chance of faster recovery and fewer complications [69]. Given these advantages, there are also potential risks to minimally invasive CABG; this review identified a slightly greater risk of bleeding and wound infection compared with open surgery. The need for patient selection and skill in minimally invasive surgery is critical to limiting these risks, even though they are inherent in all surgical procedures.

### Limitations

Some limitations of the systematic review we conducted are worth mentioning. Variability in the surgical techniques and care pathways ranged from standard open surgery to less invasive approaches. Presumably, this diversity could have influenced the pooling of results. Often, insufficient information on the exact particulars of surgical procedures, patient selection criteria, and postoperative care provided a meaningful comparison to various research studies. Some studies combined hybrid procedures or adjunctive therapies, while others omitted the details of supplementary methods that hindered the comparison between regimens.

There is room to improve the quality of these studies in future research by implementing multi-center trials and including a greater cross-section of the population. Uniform principles for assessing the surgical results and strict rules of postoperative therapy have to be followed in these studies. This standardization will improve the granularity of long-term data and help identify paradigms specific to a particular surgical strategy for CABG. Additional research is needed to determine the best practices across different patient populations.

## 5. Conclusions

This systematic study highlights that the choice of surgical strategy significantly affects the outcomes of CABG, with traditional open surgery and minimally invasive approaches showing poor compatibility. Although minimally invasive techniques hold potential, the existing information is inadequate to establish a clear preference for one method over another. To enhance our comprehension of CABG operations and improve future practice, it is imperative to conduct additional comparative clinical trials.

## Figures and Tables

**Table 1:** Comparison of Outcomes Between Open and Minimally Invasive Approaches in CABG.

Group A						
Open Approaches in CABG						
Authors	Wound infection rate	Bleeding rate	Stroke rate	Angina recurrence rate	Length of hospital stay	Mortality rate
Calin Vicol [13]	0%	2.30%	2%	27%	16 days	4%
Piroze M [14]	0.90%	1.90%	0.80%	11%	19 days	0.60%
Marco A [15]	3.10%	5.90%	5.90%	8%	8 days	8%
Gianni D [16]	25%	10.60%	1%	4.70%	38 days	5%
Pierpaolo Chivasso [17]	/	/	/	25.60%	/	7%
Ming Hao Guo [18]	47%	/	/	/	6 days	4%
Group B						
Minimally Invasive Approaches in CABG						
Authors	Wound infection rate	Bleeding rate	Stroke rate	Angina recurrence rate	Length of hospital stay	Mortality rate
Calin Vicol [13]	5%	1.70%	7%	40%	138 days	0%
Piroze M [14]	/	/	/	/	/	/
Marco A [15]	1.40%	6.00%	3.20%	6%	11 days	6.40%
Gianni D [16]	31%	11%	2%	2.30%	21 days	10%
Pierpaolo Chivasso [17]	/	/	/	11.40%	/	5.30%
Ming Hao Guo [18]	16%	/	/	/	5 days	1.30%

**Table 2:** Characteristics and Outcomes of Studies Investigating Open and Minimally Invasive Approaches in Coronary Artery Bypass Grafting.

Authors	Country	Study Design	Open CABG Patients (N)	Minimally Invasive CABG Patients (N)	Major Complications	Conclusion	Evidence Level
Calin Vicol [13]	Germany	Retrospective Cohort	45	57	Open CABG: recurrent angina (27%), bleeding (2.3%), mortality (4%). Minimally Invasive CABG: wound infection (5%), recurrent angina (40%), bleeding (1.7%).	The technical complexity of MIDCAB surpasses OPCAB, so skilled surgeons are requested.	III
Piroze M [14]	Germany	Observational	5130	0	Open CABG: arrhythmias (19.7%), wound infection (0.9%), bleeding (1.9%), mortality (0.6%).	For OPCAB, using bilateral skeletonized ITAs is safe and effective.	III
Marco A [15]	USA	Randomized trials	574	576	Open CABG: wound infection (3.1%), recurrent angina (8%), MI (5.9%), mortality (8%). Minimally Invasive CABG: wound infection (1.4%), recurrent angina (6%), MI (4.7%), mortality (6.4%).	In CABG surgeries, both open and minimally invasive procedures yield similar long-term outcomes, with the minimally invasive method offering a slight advantage in reducing wound infections.	I
Gianni D [16]	United Kingdom	Randomized trials	201	200	Open CABG: wound infection (25%), bleeding (10.6%), MI (1.0%), mortality (5.0%). Minimally Invasive CABG: wound infection (31%), bleeding (11%), MI (2.0%), mortality (10%).	OPCAB and CABG with cardiopulmonary bypass have comparable long-term health outcomes.	I
Pierpaolo Chivasso [17]	United Kingdom	Meta-analyses of RCTS	4752	2203	Open CABG: recurrent angina (25.6%), mortality (7.0%). Minimally Invasive CABG: recurrent angina (11.4%), mortality (5.3%).	The long-term benefits of off-pump CABG versus on-pump CABG remain uncertain, with existing studies showing mixed results due to methodological issues. More robust research is needed to clarify the long-term outcomes of these methods.	II
Ming Hao Guo [18]	Canada	Randomized Clinical Trial	64	450	Open CABG: None. Minimally Invasive CABG: conversion to sternotomy (3.8%), use of cardiopulmonary bypass (7.6%), mortality (1.3%).	Sternotomy CABG has a notable effect on quality of life. MICS-CABG is comparatively safer.	II
Barış Çaynak [19]	Turkey	Retrospective study	0	45	Minimally Invasive CABG: AF (11%), pleural effusion (6.7%).	The significance of employing an intraoperative flowmeter to assess the patency of the graft and the quality of the anastomosis.	III
Umberto Benedetto [20]	Germany	Retrospective post hoc RCT	923	0	Open CABG: MI (58%), mortality (5.0%).	OPCAB is linked to a higher risk of death within 3 years and a lower rate of revascularization in the coronary arteries that supply the inferolateral wall.	III
Yunpeng Ling [21]	China	Descriptive, non-experimental	0	200	Minimally Invasive CABG: AF (7.2%), mortality (0.7%).	Utilization of enhanced retractor and stabilizer in MIDCAB has the potential to yield positive results.	III
Meice Tian [22]	China	Randomized controlled trial	2655	0	Open CABG: wound infection (4.3%), recurrent angina (2.3%), bleeding (1.7%), MI (0.6%), mortality (1.1%).	Using the no-touch technique for vein graft harvesting greatly reduced the chance of vein graft occlusion and improved the patient’s prognosis.	I
Kayatta MO [23]	India	Retrospective observational	0	450	Minimally Invasive CABG: wound infection (0.9%), pneumothorax (3.1%), pleural effusion (9.1%), recurrent angina (1.6%), bleeding (1.1%), MI (1.1%).	The success of MICS-CABG relies heavily on meticulous patient selection.	III
Weimar [24]	Germany	Randomized Clinical Trial	129	0	Open CABG: MI (1%).	For patients with severe CAD undergoing CABG, combining carotid endarterectomy with CABG does not appear to offer significant advantages over isolated CABG. The study’s early termination limits the findings, but the data suggest that isolated CABG may lead to better outcomes. We need further follow-up to confirm long-term effects.	I
Marc Ruel [25]	Canada	Cohort	0	91	Minimally Invasive CABG: pleural effusion (15%), AF (17%), renal insufficiency (1.1%), wound infection (2.2%).	MICS-CABG can effectively revascularize, similar to regular CABG.	I
Shahzad G [26]	Canada	Retrospective Cohort	160	668	Open CABG: bleeding (2.5%), a wound infection (1.9%), mortality (2.5%). Minimally Invasive CABG: bleeding (3.1%), wound infection (2.4%), mortality (2.0%).	The validity and effectiveness of MIDCAB as a grafting method for the LAD artery. MIDCAB is a good alternative to traditional CABG for people with a single proximal LAD stenosis.	III
Vincenzo Giambruno [27]	Canada	Retrospective comparative analysis	546	144	Open CABG: bleeding (1.7%), MI (1.1%), stroke (2.4%), mortality (1.3%). Minimally Invasive CABG: bleeding (2.8%), MI (1.4%), stroke (2.1%).	HCR is potentially linked to a reduction in hospital mortality rates and shorter hospital stays in comparison to on-pump CABG.	III
Ali Hage [10]	USA	Retrospective comparative analysis	216	147	Open CABG: bleeding (1.5%), MI (0.5%), hemodialysis (0.5%), mortality (1.0%). Minimally Invasive CABG: bleeding (3.5%), MI (1.4%).	HCR is a viable substitute for unsuitable multivessel PCI, especially in those exhibiting a low-intermediate SYNTAX score.	III
Joseph Lamelas [28]	USA	Retrospective cohort study	0	1396	Minimally Invasive CABG: bleeding (0.8%), postoperative pacemakers (3.1%), stroke (0.8%).	Minimally invasive right thoracotomy aortic valve surgery, including both stand-alone and concurrent AVR surgeries, is a viable technique.	III
Bob Kiaii [29]	Canada	Prospective cohort	50	100	Open CABG: None. Minimally Invasive CABG: None.	The ITA can be taken out using minimally invasive videoscopic and robotic-assisted telesurgical techniques, which are both safe and effective.	II
Joshua Michael Rosenblum [7]	USA	Retrospective cohort	17411	0	Open CABG: stroke (1.3%), AF (22.5%), pneumonia (3.8%), renal failure (3.0%), mortality (13.4%).	CRV has a higher survival benefit than IRV. MA-CABG has higher survival rates than SA-CABG.	II
Alberto Repossini [30]	Italy	Retrospective analysis	0	1060	Minimally Invasive CABG: stroke (0.3%), angina (7.2%), mortality (0.8%).	MIDCAB without CPB is safe when revascularizing the LAD artery.	III
Florian Hecker [31]	Germany	Prospective study analysis	0	11	Minimally Invasive CABG: wound infection (9.1%), revascularization after surgery (18.2%).	Right-sided MIDCAB in individuals diagnosed with complicated CAD specifically affecting the RCA is safe.	II
Shameer Khubber [32]	USA	Retrospective Cohort	176	282	Open CABG: None. Minimally Invasive CABG: None.	Clinicians should take a personalized approach because the long-term risks of MACCE are similar to treating CAAs with medicine, PCI, and surgery CABG.	III
Nicholas R [33]	USA	Propensity-matched Regional Cohort	278	139	Open CABG: AF (23.4%), bleeding (2.2%), and mortality (8.3%). Minimally Invasive CABG: AF (20.1%), bleeding (2.2%), mortality (0.7%).	MICS-CABG accounted for a relatively minor proportion of the total number of CABGs conducted. MICS-CABG was linked to reduced durations of stay in the intensive care unit and hospital.	III
Jia-Ji Liu [34]	China	Retrospective, single-center, observational	0	118	Minimally Invasive CABG: None.	Performing off-pump MICS CABG to treat multi-vessel disease entails a significant learning curve. The operating length, intraoperative blood loss, ICU admission, and postoperative inpatient duration may be prolonged.	III
Hiroto Kitahara [35]	USA	Retrospective single-center	0	57	Minimally Invasive CABG: acute kidney injury (1.8%), AF (9.3%).	The process of selecting patients should incorporate a collaborative decision-making approach involving both robotic cardiac surgeons and interventional cardiologists.	III
Yugal K. Mishra [36]	India	Retrospective cohort	61	193	Open approach in CABG: bleeding (3.4%), wound infection (2%), mortality (1.7%). Minimally Invasive CABG: bleeding (1%), wound infection (1.4%).	Using the da Vinci telemanipulation system in robotically enhanced telemanipulation surgery for MIDCAB.	III
Sanin Fazlinović [37]	Sweden	Retrospective descriptive single-center cohort	421	0	Open CABG: hemorrhage (5.3%), acute renal failure (4.3%), acute respiratory failure (1%), wound infection (2.8%), mortality (2.3%).	The identification of acute surgeries as a risk factor for significant complications in the study underscores the importance of meticulous deliberation and prompt strategizing of surgical treatments, particularly in cases of acute nature.	III
Ho Young Hwang [38]	USA	Retrospective	601	300	Open CABG: bleeding (4.3%). Minimally Invasive CABG: None.	The functional importance of coronary stenosis was linked to the rates of occlusion in bypass grafts that were connected to the coronary artery five years after surgery.	III
Wenhui Gong [39]	China	Retrospective	71	61	Open CABG: wound infection (4%), MI (2%), mortality (5%). Minimally Invasive CABG: wound infection (1%), MI (3%), mortality (3%).	The RACAB grafts might be a good alternative for people who need a single or simple multi-vessel CABG.	III
Carlo Antona [40]	Italy	Preoperative study	0	41	Minimally Invasive CABG: AF (4.8%), bleeding (2%).	MIDCABG, when administered to certain individuals, exhibits reliability, safety, and promising clinical outcomes in the early and mid-term.	III
Lin Liang [41]	China	Retrospective cohort study	398	281	Open CABG: mortality (4%). Minimally Invasive CABG: MI (0.4%), mortality (2.5%).	The rates of MACCEs, such as cardiac death, MI, or recurrent revascularization, were not significantly different between CAD patients treated with MICS or CABG.	III
Zia K [42]	Pakistan	Retrospective observational study	70	30	Open CABG: renal dysfunction (1%), mortality (0.4%). Minimally Invasive CABG: renal dysfunction (2.1%).	The selection between open and minimally invasive techniques in CABG should be tailored to the individual patient, considering patient-specific features, anatomical considerations, and clinical characteristics.	III
Anno Diegeler [43]	Italy	Randomized controlled trials	0	209	Minimally Invasive CABG: graft failure (1.4%), bleeding (1.4%), MI (1.9%), mortality (0.4%).	MIDCAB is a very good way to revascularize the arteries in people with symptomatic left anterior descending coronary artery disease.	I
Paweł Bugajski [44]	Poland	Retrospective Cohort	211	0	Open CABG: recurrent angina (12%), mortality (4%).	Patients with a higher number of grafts have more time-consuming CABG procedures. These entities exhibit elevated rates of long-term mortality while maintaining comparable levels of graft patency, in-hospital mortality, and morbidity.	II
Redoy Ranjan [45]	London	Retrospective Cohort	34	34	Open CABG: bleeding (3.2%). Minimally Invasive CABG: None.	The reliability and effectiveness of CABG surgery with an endarterectomy have been established. The procedure successfully achieves the desired surgical myocardial revascularization in people with diffuse calcified CAD who do not have any other options for achieving sufficient myocardial revascularization.	III
Marek Cisowskia [46]	Poland	Retrospective study	0	50	Minimally Invasive CABG: bleeding (4.5%).	In the context of selected individuals with double-vessel CAD, hybrid procedures have been identified as both safe and successful approaches for achieving full revascularization.	III
Husam H. Balkhy [47]	Chicago	Retrospective Cohort	544	0	Open CABG: mortality (7.6%).	The robotic beating-heart TECAB is now thought to be safe and effective, with good results and similar early angiographic patency to traditional CABG surgery when performed regularly by a skilled team.	III
BH Kirmani [48]	UK	Retrospective cohort	6498	1029	Open CABG: AF (1.9%), cardiac arrest (4.7%), mortality (1.2%). Minimally Invasive CABG: AF (2.3%), cardiac arrest (2%), mortality (0.4%).	The endoscopic harvesting of saphenous veins for CABG has the same quality as open vein harvesting when it comes to long-term survival.	III
Ali İhsan Tekin [49]	Turkey	Retrospective cohort	24	23	Open CABG: None. Minimally Invasive CABG: None.	The MIDCAB technique works better than the OPCAB technique in the first few days of treatment in hospitals for patients with a serious LAD lesion.	III
Minoru Ono [50]	USA	Retrospective cohort	60	0	Open CABG: deterioration of renal function (7%), wound infection (3%), bleeding (2%), stroke (2%), mortality (5%).	Patients with functional abdominal transplants can achieve satisfactory short- and long-term outcomes with the implementation of open-heart surgery. Select patients may not require a stress-dose steroid.	III
Lufeng Zhang [51]	China	Retrospective cohort	355	300	Open CABG: None. Minimally Invasive CABG: bleeding (2%), mortality (0.3%).	The adoption of a chest wall lifting device and a redesigned stabilizer enhances the safety and ease of the MIDCAB technique. The feasibility and minimum invasiveness of the MIDCAB technique have been established as a viable alternative for individuals diagnosed with CAD.	II
Hideaki Takai [52]	Japan	Retrospective cohort study	43	0	Open CABG: arrhythmia (0.2%).	After an acute MI, OPCAB can be performed as a relatively low-risk procedure with a good death rate, even up to 14 days after the MI.	III
R Birla [53]	UK	Retrospective cohort	78	74	Open CABG: AF (1.2%), bleeding (1.3%), wound infection (2%), recurrent angina (5.1%), mortality (6.4%). Minimally Invasive CABG: AF (1.7%), bleeding (1%), wound infection (4%), recurrent angina (1.4%), mortality (1.4%).	The study revealed prospective advantages of MIDCAB, such as decreased hospitalization duration, diminished requirement for blood transfusions, and expedited recuperation.	III
Joseph T. McGinn [54]	Canada	Retrospective cohort	0	450	Minimally Invasive CABG: pleural effusion (4.1%), pneumothorax (1.4%), wound infection (1%), mortality (1.1%).	MICS CABG is a safe and effective alternative to open CABG, with promising short-term outcomes and the potential to expand access to minimally invasive heart surgery.	III
Emad A. Barsouma [55]	USA	Retrospective study	98	61	Open CABG: mortality (47.6%). Minimally Invasive CABG: mortality (19.7%).	MICS-CABG has a statistically significantly higher long-term survival rate than sternotomy-CABG in older people, even though the two procedures had some minor differences at the start.	III
G.W. Stone [56]	Poland	Randomized controlled trials	957	948	Open CABG: MI (8.3%), mortality (5.9%). Minimally Invasive CABG: MI (0.8%).	Using fluoropolymer-based cobalt-chromium everolimus-eluting stents for PCI could be a good alternative to CABG for people with left main CAD whose anatomy is not very complicated.	I
A. Diegelera [57]	Germany	Prospective study	0	271	Minimally Invasive CABG: MI (2.2%), bleeding (4.5%).	It is important to include post-operative angiographic control as part of the treatment following MIDCAB-grafting.	II
Dong Li [58]	China	Retrospective Cohort	128	32	Open CABG: MI (1.6%), mortality (1.6%). Minimally Invasive CABG: None.	The treatment options for diffuse CAD include coronary artery reconstruction and surgical patch angioplasty of the coronary artery. There was no significant difference in patient outcomes.	III
Lokeswara Rao Sajja [59]	India	Randomized controlled trials	320	0	Open CABG: mortality (1.8%).	There was no statistically significant difference between the off-pump and on-pump CABG groups in the overall rates of graft patency at 3 months when the surgery was performed by skilled surgeons who are more likely to use the off-pump method.	I
Do-Kyun Kim [60]	South Korea	Retrospective Cohort	104	0	Open CABG: MI (1.9%), mortality (17.3%).	Although the surgical death rate following urgent CABG is greater at 17.3%, a positive long-term clinical outcome can be anticipated in the event of patient survival.	III
Gursel Levent Oktar [61]	Turkey	Retrospective Cohort	737	0	Open CABG: AF (16.6%), mortality (0.1%).	Coronary artery surgery can be conducted in elderly patients with a tolerable level of risk.	III
Arani Raghavendra [62]	India	Prospective cohort	118	0	Open CABG: bleeding (6.3%).	Healthcare professionals should consider the option of on-pump CABG in cases where the preservation of graft patency is a significant issue, particularly in individuals aged 70 years and above.	II
Duk-Woo Park [63]	Korea	Retrospective Observational Cohort	1138	1102	Open CABG: MI (1.0%), mortality (0.5%). Minimally Invasive CABG: MI (8.1%), mortality (0.3%).	Patients with severe left main coronary artery (LMCA) disease can achieve revascularization through either PCI or CABG. Clinicians must consider each patient’s unique traits, preferences, and anatomical variables when deciding on the most suitable technique.	III
Issam Moussa [64]	USA	Randomized controlled trials	0	365	Minimally Invasive CABG: ventricular arrhythmia (2.3%), respiratory failure (1.7%), MI (0.6%), mortality (3.1%).	Using the MIDCAB method has led to better operator skills and stabilizing technology, which has improved the patency rate of bypass grafts.	I
Michael E. Halkos [65]	USA	Prospective cohort	0	307	Minimally Invasive CABG: stroke (0.3%), bleeding (2.3%), MI (1.6%), AF (15.3%), wound infection (2%), renal failure (2%), mortality (1.3%).	Robotic-assisted CABG is not recommended for hemodynamically unstable people who have intra-aortic balloon pumps or are having an MI that is becoming worse. There were relative contraindications for patients with a distal target vessel that was not working well or at all, who had a previous sternotomy or thoracotomy, who had a body mass index (BMI) of 40 or more, or who had a serious lung disease that made it impossible for them to maintain single-lung ventilation.	II

Abbreviations: CABG: Coronary Artery Bypass Grafting, MIDCAB: Minimally Invasive Direct Coronary Artery Bypass, OPCAB: Off-Pump Coronary Artery Bypass, ITAs: Internal Thoracic Arteries, MI: Myocardial infarction, MICS-CABG: Minimally Invasive Coronary Artery Bypass Grafting, AF: Atrial fibrillation, LAD: Left anterior descending, PCI: Percutaneous coronary intervention, HCR: Hybrid Coronary Revascularization, SYNTAX: Synergy Between PCI With Taxus and Cardiac Surgery, AVR: Aortic Valve Replacement, CRV: Complete revascularization, IRV: Incomplete revascularization, MA-CABG: Multiple arterial coronary artery bypass grafting, SA-CABG: Single-arterial coronary artery bypass grafting, CPB: Cardiopulmonary bypass, CAD: Coronary artery disease, RCA: Right coronary artery, MACCE: Major cardiovascular and cerebrovascular events, CAAs: Coronary artery aneurysms, ICU: Intensive Care Unit, RACAB: Robot-assisted coronary artery bypass grafts, TECAB: Transcatheter endoscopic aortic aneurysm bypass grafting.

## Appendix A

Retrospective and prospective cohort studies used the Newcastle Ottawa Scale for bias assessment. Using the Newcastle–Ottawa scale, case-control and cohort studies scored 7 out of 9, indicating a high level of quality. According to the level of evidence and grading recommendations of the American Society of Plastic Surgery, among the included studies, one study had a level II, four studies had a level IV, and five studies had a level V of evidence (Table A1).

**Table A1:** The Newcastle–Ottawa Scale for the included cohort prospective and retrospective studies.

	Selection				Comparability	Outcome			Quality Score	Risk of Bias (0–3: High, 4–6: Moderate, 7–9: Low)
Article	Q1	Q2	Q3	Q4	Q5	Q6	Q7	Q8		
Olson [4]	*	*	*	*	**	*	*	*	9	low
Rosenblum [7]	*	*	*	*	**	*	*	*	9	low
Vicol [13]	*	*	*	*	**	*	*	*	9	low
Barış Çaynak [19]	*		*			*		*	4	moderate
Umberto Benedetto [20]	*	*	*	*	**	*	*	*	9	low
Yunpeng Ling [21]	*		*	*			*	*	5	moderate
Kayatta MO [23]		*	*			*	*		4	moderate
Marc Ruel [25]	*		*	*	*	*	*	*	7	low
Shahzad G [26]	*	*	*	*	**	*	*	*	9	low
Vincenzo Giambruno [27]	*	*	*	*	**		*	*	8	low
Ali Hage [10]	*	*	*	*	**	*	*	*	9	low
Joseph Lamelas [28]	*		*	*	*	*	*	*	7	low
Bob Kiaii [29]	*		*	*	**	*	*	*	8	low
Joshua Michael Rosenblum [7]	*	*		*	**	*	*	*	8	low
Alberto Repossini [30]	*	*	*	*		*	*	*	7	low
Florian Hecker [31]	*		*	*	**	*	*	*	8	low
Shameer Khubber [32]	*		*	*	*	*	*	*	7	low
Nicholas R [33]	*	*	*	*	**	*	*	*	9	low
Jia-Ji Liu [34]	*		*	*	**	*	*	*	8	low
Hiroto Kitahara [35]	*	*	*	*	**	*	*	*	9	low
Yugal K. Mishra [36]	*	*	*	*	**	*	*	*	9	low
Sanin Fazlinović [37]	*	*	*	*	**	*	*	*	9	low
Ho Young Hwang [38]	*		*	*	*	*	*	*	7	low
Wenhui Gong [39]	*		*	*	*	*	*	*	7	low
Carlo Antona [40]	*	*	*	*	**	*	*	*	9	low
Lin Liang [41]	*		*	*		*	*	*	6	moderate
Zia K [42]	*	*	*	*	*	*	*	*	8	low
Paweł Bugajski [44]	*	*	*	*	*	*	*	*	8	low
Redoy Ranjan [45]	*		*	*	*	*	*	*	7	low
Marek Cisowskia [46]	*	*	*	*	*	*	*	*	8	low
Husam H. Balkhy [47]	*	*	*	*	**	*	*	*	9	low
BH Kirmani [48]	*	*	*	*	**	*	*	*	9	low
Ali İhsan Tekin [49]	*	*	*	*	*	*	*	*	8	low
Minoru Ono [50]	*	*	*	*	*	*	*	*	7	low
Lufeng Zhang [51]	*	*	*	*	**	*	*	*	8	low
Hideaki Takai [52]	*	*	*	*	*	*	*	*	7	low
Rashmi Birla [53]	*	*	*	*	**	*	*	*	9	low
Joseph McGinn [54]	*	*	*	*	**	*	*	*	9	low
Emad Barsoum [55]	*	*	*	*	*	*	*	*	8	low
Diegeler [57]	*	*	*	*	**	*	*	*	8	low
Dong Li [58]	*	*	*	*	**	*	*	*	9	low
Do-Kyun Kim [60]	*	*	*	*	**	*	*	*	8	low
Oktar [61]	*	*	*	*	**	*	*	*	9	low
Raghuram [62]	*	*	*	*	**	*	*	*	8	low
Park [63]	*	*	*	*	**	*	*	*	9	low
Halkos, 2014 [65]	*	*	*	*	**	*	*	*	8	low

**Selection:** Q1. Representativeness of the exposure cohort? Q2. Selection of the non-exposure cohort? Q3. Ascertainment of exposure? Q4. Demonstration that outcome of interest was not present at the start of the study?; **Comparability:** Q5. Comparability of cohort based on the design or analysis?; **Outcome: Q6**. Assessment of outcome? Q7. Was follow-up long enough for outcomes to occur? Q8. Adequacy of follow-up of cohorts?; *: means the corresponding quality component or criterion is partially met, **: means the corresponding quality component or criterion is fully met.

## Appendix B

**Table A2:** Bias of the included cohort prospective and retrospective studies evaluated according to the Newcastle–Ottawa Scale.

Author	Bias Arising from the Randomization Process	Bias Due to Deviations from Intended Interventions	Bias Due to Missing Outcome Data	Bias in Measurement of the Outcome	Bias in Selection of the Reported Result	Overall RoB
Taggart [1]	low	some concerns	low	low	low	low
Shroyer [6]	low	some concerns	low	low	low	low
Zenati, Marco [15]	low	some concerns	low	low	low	low
Angelini [16]	low	some concerns	low	low	low	low
Ming Hao Guo [18]	low	some concerns	low	low	low	low
Meice Tian [22]	low	some concerns	low	low	low	low
Weimar [24]	low	Some Concerns	low	low	Some Concerns	Some Concerns
Anno Diegeler [43]	low	some concerns	some concerns	low	some concerns	low
Stone [56]	low	Low	high	low	low	low
Sajja [59]	low	Low	Some Concerns	Low	Some Concerns	Some Concerns
Moussa [64]	low	Some Concerns	Some Concerns	Low	Some Concerns	Some Concerns

## Appendix C

The MINORS tool was used to assess the quality of the non-randomized studies included in this review. The total score ranged from 6 to 18, with a mean score of 12.3. The items with the lowest scores were the prospective calculation of the study size (score of 0 in all studies), the unbiased assessment of the study endpoint (score of 0 in all studies), and the inclusion of a consecutive series of patients (score of 1 or 2 in most studies). The items with the highest scores were the clearly stated aim of the study (score of 2 in all studies), the description of patient characteristics (score of 2 in all studies), and the clearly defined endpoints (score of 2 in all studies) (Table A3).

**Table A3:** The Methodological Index for Non-Randomized Studies (MINORS).

Item	Bansal 2023	Ullah 2023	Benedetto 2009	Kikuchi 2017	Parnell 2018	Hage 2019	Ouzzani 2016	Davierwala 2018	Chivasso 2016
A clearly stated aim	2	2	2	2	2	2	2	2	2
Inclusion of consecutive patients	0	2	2	0	0	2	0	2	2
Prospective collection of data	0	2	1	0	0	2	0	2	1
Endpoints appropriate to the aim of the study	2	2	2	2	2	2	2	2	2
Unbiased assessment of the study endpoint	1	1	2	1	1	2	2	2	1
Follow-up period appropriate to the aim of the study	0	2	2	2	0	2	0	2	2
Loss to follow-up less than 5%	0	1	1	0	0	1	0	1	1
Prospective calculation of the study size	0	1	1	0	0	1	0	1	1
An adequate control group	0	1	2	1	0	2	0	1	2
Contemporary groups	0	1	2	1	0	2	0	1	2
Baseline equivalence of groups	0	1	2	1	0	2	0	1	2
Adequate statistical analyses	2	2	2	2	2	2	2	2	2
Total Score	7	17	19	11	7	20	8	17	18

## Appendix D

**Table A4:** Systematic Review Protocol and Support Template.

Title of the Systematic Review	Comparative the Effectiveness of Open and Minimally Invasive Approaches in Coronary Artery Bypass Grafting: A Systematic Review
Author and Affiliations	Arwa Tawfiq Alsharif College of Medicine and Surgery, Batterjee Medical College, Jeddah, Saudi Arabia 140088.arwa@bmc.edu.sa Abdulaziz Tawfiq Alsharif College of Medicine and Surgery, Vision College, Jeddah, Saudi Arabia 202313034@vision.edu.sa Ghadah Dhafer Alshamrani College of Medicine and Surgery, Batterjee Medical College, Jeddah, Saudi Arabia 140065.ghadah@bmc.edu.sa Abdulhameed Luay Abu Alsoud College of Medicine and Surgery, Batterjee Medical College, Jeddah, Saudi Arabia 140011.abdulhameed@bmc.edu.sa Rowaida Abdullah Durian College of Medicine and Surgery, Batterjee Medical College, Jeddah, Saudi Arabia 140238.rowaida@bmc.edu.sa Sarah Mohammad Aljohani College of Medicine and Surgery, Batterjee Medical College, Jeddah, Saudi Arabia 140321.sarah@bmc.edu.sa Hawazen Omar Alahmadi Faculty of Medicine, Taibah University, Al-Madinah Almunawwarah, Saudi Arabia Tu4354677@taibahu.edu.sa Samratul Fuadah College of Medicine and Surgery, Batterjee Medical College, Jeddah, Saudi Arabia 140034.samratul@bmc.edu.sa Atheer Mohammed Mohammed College of Medicine and Surgery, Batterjee Medical College, Jeddah, Saudi Arabia 140278.atheer@bmc.edu.sa
Supervisor/Project PI	Fatma E. Hassan Department of Medical Physiology, Batterjee Medical College, Jeddah 21442, Saudi Arabia Fatma.hassan@bmc.edu.sa
(1) Background of the systemic review	
Coronary artery bypass grafting (CABG) is the most effective surgical procedure for coronary artery disease and the leading global cause of morbidity and mortality. Most notably, open and minimally invasive CABG approaches have evolved with advances in surgical techniques and technology. The advantages and disadvantages of each procedure link to the differences between these two distinct surgical methods [70]. For years, CABG, an open-heart surgery, has been the primary method of coronary revascularization, requiring a sternotomy with cardiopulmonary bypass (CPB) on a heart-lung machine. This ensures complete revascularization and provides the surgeon with direct access to each of the coronary vessels. This technique has two major disadvantages: high rates of perioperative morbidity and long convalescent periods. They often experience longer in-hospital healthcare episodes followed by a prolonged recovery time, which may decrease their quality of life [71]. Conversely, the minimally invasive CABG procedures have emerged as a promising alternative. These approaches reduced the size and number of incisions required, resulting in reduced surgical trauma and, consequently, improved post-surgical recovery. Minimally invasive CABG comprises robot-assisted surgery, grafts with completely endoscopic treatments or thoracotomy incisions, and other techniques. The advocates of minimally invasive CABG contend that it offers the potential for reduced pain, a faster return to regular activities with shorter hospital stays, and better cosmetic results [72]. Whether open or minimally invasive CABG should be the surgical treatment depends on the patient, surgeon, and institution. However, the medical community continues to grapple with this issue. Although some research states that minimally invasive CABG similar to OPCAB could give similar or better results in mortality, morbidity, and long-term graft patency, others say open CABG has remained the gold standard due to its established record of outcomes and capacity for full revascularization [71,73].	
(2) Aim of the systemic review	
This systematic review aims to evaluate and compare the effectiveness of open and minimally invasive approaches in Coronary Artery Bypass Grafting (CABG) procedures.	
(3) Specific objectives of the systemic review	
- To compare the safety profiles of open and minimally invasive CABG procedures. - To analyze resource utilization aspects, including length of hospital stay, and post-surgical complication associated with each surgical approach. - To assess and compare the long-term outcomes, associated with open and minimally invasive CABG.	
(4) Criteria for including studies in this review	
Population, or participants and condition of interest	This includes patients of different ages, and genders, who have been diagnosed with coronary artery disease (CAD) in various healthcare settings. The condition of interest for this systematic review research is to investigate and compare the outcomes and effectiveness of open and minimally invasive approaches in CABG procedures.
Interventions or exposures	- Open Coronary Artery Bypass Grafting (Open CABG): This intervention involves the conventional surgical approach for coronary revascularization, which generally includes a sternotomy and the use of a heart-lung machine for cardiopulmonary bypass. This review will assess their collective effectiveness. - Minimally Invasive Coronary Artery Bypass Grafting (Minimally Invasive CABG): This intervention encompasses a range of minimally invasive surgical techniques for coronary revascularization, such as small thoracotomy incisions, robot-assisted surgery, and totally endoscopic procedures. This review will examine the comparative effectiveness of these approaches in terms of clinical outcomes, and safety.
Comparisons or control groups	- Open CABG as Control: This group comprises patients who have undergone the traditional open CABG procedure. These patients serve as the control group against which the outcomes of other interventions, such as minimally invasive CABG, will be compared. - Minimally Invasive CABG as Comparisons: In certain analyses, patients who have undergone minimally invasive CABG may serve as a comparative assessment of the relative benefits and drawbacks of each surgical approach.
Outcomes of interest	Surgical complications (Wound infection rate, Bleeding rate, Stroke rate, Angina recurrence rate, Length of hospital stay, and Mortality rate)
Setting	- Hospitals - Outpatient clinics - Cardiac surgical centers
Inclusion criteria	Studies that of the evaluate the open approaches in coronary artery bypass grafting. Studies that of the evaluate the minimally invasive approaches in coronary artery bypass grafting. Studies involving patients diagnosed with coronary artery disease who require coronary artery bypass grafting. Randomized controlled trials (RCTs), quasi-experimental studies, cohort studies, case-control studies, and observational studies. Studies published in peer-reviewed journals or other credible sources. Studies published in the English language.
Exclusion criteria	Studies that focus on other surgical procedures or interventions unrelated to coronary artery bypass grafting. Case-report studies, Review articles Animal studies, in vitro studies, and review articles. Studies published in languages other than English. Studies with insufficient data or lack of relevant outcome measures.
(5) Search methods	
Electronic databases	- PubMed database - MEDLINE database - Web of science
Keywords	(Coronary Artery Bypass Grafting OR Coronary Artery Bypass Surgery OR Coronary Artery Bypass OR Aortocoronary Bypass OR Bypass Surgery OR Coronary Artery Disease OR Aortocoronary OR CABG OR Open-heart surgery OR Minimally invasive surgery OR Endoscopic Surgery OR Surgical outcomes OR Mortality OR Graft patency OR Graft Occlusion OR Vascular Graft Restenosis OR Length of hospital stay)
(6) Methods of review	
Details of methods, number of reviewers, how agreements are to be reached and disagreements dealt with, etc.	Four main reviewers and a fifth to resolve any disagreements. Resolving any outstanding disagreements, an article by Dr. Fatma and Dr. Arwa.
Quality assessment tools or checklists used with references or URLs	Grading of Recommendations Assessment, Development, and Evaluation (GRADE) system to evaluate the overall quality of evidence and strength of recommendations. Newcastle–Ottawa Scale (NOS): to assess the quality of non-randomized studies, such as cohort and case-control studies. Revised Cochrane Risk-of-Bias Tool (RoB 2) to evaluate the risk of bias in randomized controlled trials (RCTs). MINORS tool: to assess the methodological quality of non-randomized studies, including case series.
Narrative synthesis details of what and how synthesis will be performed	A narrative synthesis will be conducted alongside any meta-analysis and will be carried out using a framework that consists of four elements: 1—Developing a theory of how the intervention works, why, and for whom. 2—Developing a preliminary synthesis of findings of included studies. 3—Exploring relationships within and between studies. 4—Assessing the robustness of the synthesis.
Meta-analysis details of what and how analysis and testing will be performed. If no meta-analysis is to be conducted, please give a reason.	Although a meta-analysis is planned, this will only become apparent when we see what data has been extracted and made available from the systematic review. Need to think about how heterogeneity will be explored.
Grading evidence system used, if any, such as GRADE	GRADE will be used for evidence assessment.
(7) Presentation of results	
Additional material summary tables, flowcharts, etc, to be included in the final paper	A flow chart of the whole process Protocol Data extraction from and tables Forest plots of studies are included in the final review.

## Appendix E

**Table A5:** PRISMA 2020 Checklist.

Section and Topic	Item #	Checklist Item	Location where Item Is Reported
**Title**			
Title	1	Identify the report as a systematic review.	Page 1; Lines 3 and 4
**Abstract**			
Abstract	2	See the PRISMA 2020 for Abstracts checklist.	Page 1; Lines 24 to 39
**Introduction**			
Rationale	3	Describe the rationale for this review in the context of existing knowledge.	Page 2; Lines 54 to 64
Objectives	4	Provide an explicit statement of the objective(s) or question(s) this review addresses.	Page 2; Lines 75 to 86
**Methods**			
Eligibility criteria	5	Specify the inclusion and exclusion criteria for this review and how studies were grouped for the syntheses.	Page 3; Lines 112 to 126
Information sources	6	Specify all databases, registers, websites, organizations, reference lists and other sources searched or consulted to identify studies. Specify the date when each source was last searched or consulted.	Page 2 and 3; Lines 90 to 110
Search strategy	7	Present the full search strategies for all databases, registers and websites, including any filters and limits used.	Page 2 and 3; Lines 90 to 110
Selection process	8	Specify the methods used to decide whether a study met the inclusion criteria of this review, including how many reviewers screened each record and each report retrieved, whether they worked independently, and if applicable, details of automation tools used in the process.	Page 3; Lines 128 to 139
Data collection process	9	Specify the methods used to collect data from reports, including how many reviewers collected data from each report, whether they worked independently, any processes for obtaining or confirming data from study investigators, and if applicable, details of automation tools used in the process.	Page 3; Lines 128 to 139
Data items	10a	List and define all outcomes for which data were sought. Specify whether all results that were compatible with each outcome domain in each study were sought (e.g., for all measures, time points, analyses), and if not, the methods used to decide which results to collect.	Page 4; Lines 153 to 165
	10b	List and define all other variables for which data were sought (e.g., participant and intervention characteristics, funding sources). Describe any assumptions made about any missing or unclear information.	Page 4; Lines 153 to 165
Study risk of bias assessment	11	Specify the methods used to assess risk of bias in the included studies, including details of the tool(s) used, how many reviewers assessed each study and whether they worked independently, and if applicable, details of automation tools used in the process.	Page 4; Lines 141 to 151
Effect measures	12	Specify for each outcome the effect measure(s) (e.g., risk ratio, mean difference) used in the synthesis or presentation of results.	ND
Synthesis methods	13a	Describe the processes used to decide which studies were eligible for each synthesis (e.g., tabulating the study intervention characteristics and comparing against the planned groups for each synthesis (item #5)).	ND
	13b	Describe any methods required to prepare the data for presentation or synthesis, such as handling of missing summary statistics, or data conversions.	ND
	13c	Describe any methods used to tabulate or visually display results of individual studies and syntheses.	ND
	13d	Describe any methods used to synthesize results and provide a rationale for the choice(s). If meta-analysis was performed, describe the model(s), method(s) to identify the presence and extent of statistical heterogeneity, and software package(s) used.	Page 4; Lines 153 to 161
	13e	Describe any methods used to explore possible causes of heterogeneity among study results (e.g., subgroup analysis, meta-regression).	Page 4; Lines 153 to 161
	13f	Describe any sensitivity analyses conducted to assess robustness of the synthesized results.	ND
Reporting bias assessment	14	Describe any methods used to assess risk of bias due to missing results in a synthesis (arising from reporting biases).	Page 4; Lines 141 to 151
Certainty assessment	15	Describe any methods used to assess certainty (or confidence) in the body of evidence for an outcome.	ND
**Results**			
Study selection	16a	Describe the results of the search and selection process, from the number of records identified in the search to the number of studies included in this review, ideally using a flow diagram.	Page 5; Lines 167 to 183
	16b	Cite studies that might appear to meet the inclusion criteria, but which were excluded, and explain why they were excluded.	Page 5; Lines 168 to 174
Study characteristics	17	Cite each included study and present its characteristics.	Page 6 to 14; Lines 184 to 217
Risk of bias in studies	18	Present assessments of risk of bias for each included study.	Page 17 to 23; Lines 333 to 356
Results of individual studies	19	For all outcomes, present, for each study: (a) summary statistics for each group (where appropriate) and (b) an effect estimate and its precision (e.g., confidence/credible interval), ideally using structured tables or plots.	Page 6 to 14; Lines 213 to 217
Results of syntheses	20a	For each synthesis, briefly summarise the characteristics and risk of bias among contributing studies.	Page 17 to 23; Lines 333 to 356
	20b	Present results of all statistical syntheses conducted. If meta-analysis was conducted, present for each the summary estimate and its precision (e.g., confidence/credible interval) and measures of statistical heterogeneity. If comparing groups, describe the direction of the effect.	ND
	20c	Present results of all investigations of possible causes of heterogeneity among study results.	ND
	20d	Present results of all sensitivity analyses conducted to assess the robustness of the synthesized results.	ND
Reporting biases	21	Present assessments of risk of bias due to missing results (arising from reporting biases) for each synthesis assessed.	ND
Certainty of evidence	22	Present assessments of certainty (or confidence) in the body of evidence for each outcome assessed.	ND
Discussion			
**Discussion**	23a	Provide a general interpretation of the results in the context of other evidence.	Page 15; Lines 260 to 289
	23b	Discuss any limitations of the evidence included in this review.	Page 16; Lines 291 to 298
	23c	Discuss any limitations of this review process used.	Page 16; Lines 291 to 298
	23d	Discuss the implications of the results for practice, policy, and future research.	Page 16; Lines 299 to 305
**Other information**			
Registration and protocol	24a	Provide registration information for this review, including register name and registration number, or state that this review was not registered.	Page 2; Lines 90 to 92
	24b	Indicate where this review protocol can be accessed, or state that a protocol was not prepared.	Page 24; Lines 359 to 362
	24c	Describe and explain any amendments to information provided at registration or in the protocol.	Page 24; Lines 359 to 362
Support	25	Describe sources of financial or non-financial support for this review and the role of the funders or sponsors in this review.	Page 17; Line 320
Competing interests	26	Declare any competing interests of review authors.	Page 17; Line 332
Availability of data, code, and other materials	27	Report which of the following are publicly available and where they can be found: template data collection forms; data extracted from included studies; data used for all analyses; analytic code; any other materials used in this review.	Pages 17 to 27; Lines 333 to 362
